# Physical influences on seafarers are different during their voyage episodes of port stay, river passage and sea passage: A maritime field study

**DOI:** 10.1371/journal.pone.0231309

**Published:** 2020-04-08

**Authors:** Marcus Oldenburg, Christian Felten, Jörg Hedtmann, Hans-Joachim Jensen

**Affiliations:** 1 Institute for Occupational and Maritime Medicine Hamburg (ZfAM), University Medical Center Hamburg-Eppendorf (UKE), Hamburg, Germany; 2 BG Verkehr (German BG for transport and traffic), Hamburg, Germany; 3 Flensburg University of Applied Sciences, Flensburg, Germany; Universidade Federal de Minas Gerais, BRAZIL

## Abstract

**Introduction:**

During a sea voyage, crew members of vessels are permanently exposed to physical stress caused by noise, vibration and heat. This study aims to describe the extent of the physical influences on board container ships and the resulting stress.

**Methods:**

Up to four scientific investigators accompanied six sea voyages on container ships under German management. Workplace and person-related measurements for noise, vibration and climatic parameters were carried out on the vessels during the three different voyage episodes (port stay, river passage and sea passage).

**Results:**

The interviewed seafarers reported, in decreasing order, the level of psychological stress due to vibration (80.6%), noise (71.8%) and, much less, heat (45.7%) in the workplace.

In terms of workplace-related physical measurements, the highest noise levels were found in the engine room (104 dB (A)), in the workshop (81 dB (A)) and on deck (77 dB (A)), irrespective of the voyage episode. Some noise measurements in the recreational area revealed levels above the threshold. All measured 180 vibration values were below the relevant threshold limits—with the highest values in the engine room (62 mm/s^2^), followed by the workshop (37 mm/s^2^) and the engine control room (34 mm/s^2^). In terms of thermal comfort, none of the measured climatic parameters differed significantly between the voyage episodes. According to the person-related physical measurements, the noise exposure was particularly pronounced among the engine room personnel with an average noise level of 96 dB (A) (often during cleaning, painting and repair work). In contrast, the deck crew and nautical officers were respectively exposed to an average level of 83 dB (A) and 77 dB (A) at work.

**Discussion:**

A relevant stress level due to physical loads was detectable in the present study. As ship crews are exposed to the physical influences on board for 7 days a week over several months, further research is recommended to assess the long-term health effects for seafarers.

## Introduction

The search for ever faster and more economical ships is difficult to reconcile with the demand for a quiet, low-vibration and well air-conditioned working and living environment on board. Physical stress, such as noise, vibration and heat, sometimes elicits considerable strain on the shipboard crew [1–3]. Several previous maritime studies have shown that these variables are the main physical influences that can affect the well-being of seafarers [1,2]. Thus, it is important to evaluate the level of these physical influences in different locations on board and during different job activities.

The monotonous noise of the vessel’s engine, the smooth ship’s vibrations and the continuous slow ship’s movements (during calm weather conditions) can lead to sleepiness of the shipboard crew. Higher levels of exposition to noise and vibrations can also increase sleep problems and poorer sleep quality [4]. It has also been described that sudden noise evoked by the handling of containers in harbors can interrupt sleep periods on board [5].

In particular, sound pressure levels above 60 dB (A) (a weighted level to account for the relative loudness perceived by the human ear) and severe ship vibration can cause sleep disturbances in terms of reduced sleep duration, decrease in REM periods, and repeated waking episodes [6,7]. Cumulative effects are also known to occur that can lead to physical and mental fatigue [8,9]. Additionally, high noise levels above 85 dB (A) can cause, among other things, hearing loss [10].

High noise levels on board are caused by ship operation, loading and unloading (especially on container ships) − during port stay −, port facilities (cranes, for example) as well as environmental noise (wind and sea) [11,12]. At the workplace, particularly high noise levels are expected in the engine room and, in declining order, in the engine control room and other areas with increasing distance from the ship’s engine. Furthermore, some job-related tasks produce higher noise levels (e.g. repair work on the engine, manual work on board such as knocking out rust).

Strong vibration can also reduce the well-being of ship crews (e.g. seasickness) [13,14] or even result in health impairment (e.g. lower back pain, abnormalities in intervertebral discs) [15–17].

A heat effect at the workplace is particularly present in the engine room and in the galley. Young and previously healthy seamen can be affected by heat cramps, heat collapse and even fatal heat strokes [18,19]. This is especially the case among crew members when working in the engine room or in the sun on deck [20]. Good heat insulation and the use of an efficient air conditioning system can significantly reduce the heat load in the engine room and adjacent rooms. Thus, it is also assumed that the physical influences due to climatic parameters differ between the engine room, the engine control room and other air-conditioned areas far away from the engine room.

In total, physical influences can affect the well-being and health of seafarers, who are usually on board for several months at a time. As the crew may only have the opportunity to leave their ship for a short while during port stays, they are usually exposed to physical influences on board during their working and leisure time for months [21].

Overall, the stress on shipping crews caused by physical parameters substantially depends on the voyage episode of the ship (port stay, river or sea passage). The port stay comprises the time from the arrival to the departure of the ship. The river passage of the seagoing vessel takes place on a river or canal to and from the port into the open sea, and the sea voyage begins after the end of the river passage and ends with the start of the following river passage to reach the port. A recent previous study revealed significant differences, particularly concerning the strain on seafarers during the three voyage episodes (port stay, river passage and sea passage) based on measurements of the crews’ energy expenditure and heart rate variability [22]. However, a differentiation of stress levels and measured physical influences related to the various voyage periods has never been made up to now. Therefore, the extent of physical influences on board of container ships and the resulting stress on the crews were analyzed in this study, taking into account the voyage episodes and the occupational groups.

## Methods

Up to 4 scientific researchers accompanied 6 sea voyages (4 small container ships in the exclusive coastal area and 2 large container ships with worldwide shipping routes) on ships under German management in the North and Baltic Sea (including the English Channel) respectively in a comparable coastal operation with a high port frequency.

### Study sample

Of the 112 sailors aboard the 6 container ships, 104 sailors participated in this study (participation rate 92.9%). The participation in the study was completely voluntary and the data collected was pseudonymized. The study was approved by the Ethics Committee of the Hamburg Medical Association (no PV4395). All participants gave their written informed consent before taking part in this study.

The study collective consisted of 19 nautical officers, 51 deck ratings and 34 employees from the engine room. The average age of the exclusively male crew members was 35.4 years (20–62 years).

### Investigated vessels

Three of the 6 selected ships were under German flag, one under English flag, one under that of Antigua-Barbuda and one Liberia (Table 1). The average year of construction of the ships was 2007 (1998–2010). At the time of the investigations, the vessels had an average age of 5.4 years (4–14 years).

**Table 1:** Description of the investigated vessels.

Ships	Flag	Year of construction	Gross tonnage	TEU^b^	Max velocity	Total length	Width	Crew members participating
			*GRT*^a^	*n*	*kn*	*m*	*m*	*n*
**1**	Antigua- Barbuda	2010	9,983	877	18.3	141	24	16
**2**	Germany	2006	91,649	8,204	24.4	333	42	26
**3**	Germany	2008	73,899	5,905	24.7	286	31	24
**4**	England	2003	7,519	822	16.7	137	22	13
**5**	Germany	1998	5,056	648	17.0	118	18	12
**6**	Liberia	2009	15,739	1,421	18.2	168	27	13

^a^GRT: gross registered ton

^b^TEU: twenty-foot equivalent unit

Four ships were feeder ships, i.e. smaller container ships that operated as suppliers and distributors for large seagoing ships and seaports. They had an average gross tonnage (GT) of 8,751 and a mean length of 139 m (Table 1). The 2nd and 3rd ships were large container vessels with a gross tonnage of more than 70,000 (length> 280 m), which were only temporarily used in the North Sea feeder service. The investigated container ships were representative in size and age for the German container fleet.

### Stress of volunteers

A questionnaire determined the subjective assessment of stress of all crew members on board related to the physical environmental influences (noise, vibration and heat). The seafarers rated in a bivariate manner (yes/no) whether they normally experience the various physical factors on board as stressful. Furthermore, the seafarers had the option to comment the physical influences during their stay on board in free text. This questionnaire was tested and used in several pilot studies in the specific maritime context [1].

In the present study, a psychologically trained interviewer with seafaring experience applied this questionnaire in the form of a standardized interview on board before the start of the physical measurements. As a quality criterion, questioning by an interviewer generally enables an immediate completeness check and the elimination of ambiguities [23,24].

### Physical measurements

Measuring technicians from BG Verkehr carried out objective measurements of noise, vibration and climatic parameters in predefined working and living areas on board of the 6 container ships.

### Noise

The International Convention for the Safety of Life at Sea (SOLAS), 1974, must be considered regarding the regulations that shipbuilders must adhere to concerning noise. The Code on Noise Levels On Board Ships (MSC 91, from 2012) [25] adopted a new SOLAS regulation II-1/3-12 to require new ships (keel laid since 2014) to be constructed in a way that reduces on-board noise and protects personnel from noise. This Code sets out mandatory maximum noise level limits for machinery spaces, control rooms, workshops, accommodation and other spaces on board ships.

In accordance with the Technical Rules for the Noise and Vibration Industrial Safety Ordinance, the average sound pressure level (LAeq, A-weighted equivalent continuous sound level) was determined using personal and location-related measuring methods. Personal measurements were taken with a sound dosimeter (Type 4445 (class 2) from Brüel & Kjaer, Nærum, Denmark), and a calibrator type (4231 (class 1)) was used to calibrate the instruments. Person-related measurements were only determined for noise because specific job-related tasks were likely to produce higher noise levels (e.g. repair work on the engine, manual work on board such as knocking out rust) − in contrast to vibration and climatic parameters, which are not associated with the seafarers’ tasks.

The location-related measurements were carried out in accordance with DIN ISO 2923 with a sound level meter (type 2250 (class 1) from Brüel & Kjaer, Nærum, Denmark) (2923:2003–03 2003).

The on-site noise exposure on board was recorded during the various voyage episodes in the following working / living areas (two-minute measurement period in each case): bridge, office, deck, engine room, engine control room, workshop, rating mess room, galley, cabin and recreational room.

### Vibration

According to DIN ISO 6954 (6954:2000 2000), as a guide, the limit values are, for example, 140 mm/ s^2^ for the bridge area, 221 mm/ s^2^ for the engine room and 112 mm/ s^2^ for the living space. The vibration measurements were carried out with a vibration measuring device (Norsonic 136, Oelde-Stromberg, Germany), with which whole-body vibration was measured. The vibration measurement was carried out in all three axes, with the vertical axis often showing the strongest amplitude. In accordance with DIN ISO 6954, only the largest amplitude of the three measured axes was taken into account in the evaluation.

In this study, vibration on board was recorded during the three voyage episodes. The vibration measurements were carried out in the same working / living areas where the local noise measurements took place (see above).

### Climatic parameters and thermal comfort

Thermal comfort was estimated from calculations performed by the Almemo system (Ahlborn, Germany), with which the air, radiation and wet temperatures, air velocity and relative humidity were recorded. In combination with the additionally recorded parameters clothing factor, activity level and mechanical performance, the Predicted Mean Vote (PMV) and the Predicted Percentage of Dissatisfied (PPD) were calculated according to DIN EN ISO 7730 (7730 2005) [26]. The PMV is an index that forecasts the average thermal comfort assessment score from -3.0 (cold) to +3 (hot) on a 7-level thermal comfort rating scale. The PPD value can be calculated on the basis of the PMV value [27,28].

The work-related climatic parameters analyzed were mainly dependent on the heat produced by the ship’s engine. Therefore, differences in the heat exposure are assumed between the engine room, the adjacent engine control room and an air-conditioned reference area far away from the engine room (galley). Consequently, in this study, the thermal comfort and indoor climatic parameters (temperature, air velocity and relative humidity) were measured in these work areas during the three voyage episodes.

### Statistical analysis

Data analysis was performed with SPSS for Windows (version 20.0, SPSS GmbH Software, Munich, Germany). The perception of stress was expressed as percentage. Continuous variables were presented as median (min-max). All physical data measured were not normally distributed. In cases of higher differences (> 10%) of two physical data between the voyage periods, the non-parametric Kruskal-Wallis test and the post-hoc analysis were applied to compare different groups. All indicated p-values were two-sided and a p-value of <0.05 was regarded as statistically significant.

## Results

According to subjective estimation, the seafarers reported psychological stress due to vibration (80.6%), noise (71.8%) and much less to heat (45.7%) in the workplace. Stress due to noise exposure was particularly frequent among engine room personnel (83.7%) and significantly less often among deck crew (65.4%) (p = 0.009). In the free text, several crew members stated significant sleep problems in port caused by noisy container handling and clearly noticeable ship movement.

### Workplace-related measurements in different voyage episodes

As part of the physical location measurements, the highest noise level was in the engine room at up to 110 dB (A), in the workshop (up to 87 dB (A) during river and seafaring passage) and on deck (up to 83 dB (A)) (Table 2). Thanks to an obviously good sound insulation, only maximum noise levels up to 79 dB (A) were recorded in the engine control room. Overall, assuming a corresponding theoretical exposure over eight hours per day, it is expected that some measurements exceed the lower and partly also the upper threshold in some work areas on board. In the recreational areas (crew mess room), a maximum of 71 dB (A) was achieved. In the sleeping area (cabin), the average noise level was 57 dB (A) (range 36–66 dB (A)).

**Table 2:** Noise on board depending on the different voyage periods.

Physical influences	Location	*General*	Voyage period		
			*Port stay*	*River passage*	*Sea passage*
**LAeq**, dB (A) median (min-max)^a^	Bridge	57 (45–73)	67 (45–73)	67 (57–73)	57 (49–67)*^#^
	Office	62 (40–69)	59 (40–64)	64 (57–69)	62 (53–66)
	Deck	77 (62–83)	75 (62–80)	77 (47–81)	78 (62–83)
	Engine room	104 (98–110)	104 (99–108)	106 (99–110)	102 (98–105)
	Engine control room	72 (56–79)	68 (56–74)	72 (61–78)	73 (63–79)
	Workshop	81 (65–87)	76 (65–81)	84 (68–87)*	81 (70–86)
	Crew mess room	63 (46–71)	56 (46–59)	66 (51–71)*	64 (53–68)
	Galley	68 (57–73)	67 (59–71)	69 (62–73)	66 (57–71)
	Cabin	57 (36–66)^&^	49 (36–53)	60 (51–66)*	57 (47–63)*
	Recreational room	62 (53–66)	62 (53–65)	57 (55–58)	63 (54–66)

^&^significant Kruskal-Wallis test in relation to port stay,

*different from “port stay”,

^#^different from “river passage”

^a^min-max = min to max LAeq (time-averaged sound pressure level with A-frequency weighting)

There was no difference between the three voyage episodes in the average noise exposure in the area of the bridge, office, deck, engine room, engine control room, workshop, crew mess room, galley and recreational room. During port stay, particularly high noise levels were observed in the engine room (on average 104 dB (A) (range 99–108 dB (A)) and in the engine control room (average 68 dB (A) (range 56–74 dB (A)) (Table 2). Significantly lower noise levels were measured in the cabin during port stay than during river and sea passage. In post-hoc analyses, the noise was significantly lower in the workshop and the crew mess room during port stay compared to river passage. The noise on the bridge was significantly lower during sea passage in comparison to river passage or port stay.

With regard to the vibration measurements, all measured values were below the threshold values of DIN ISO 6954 for permissible mechanical vibration on seagoing vessels. The highest vibration was measured, in declining order, in the engine room, the workshop, the engine control room, on the bridge and in the office (Table 3). Vibration was distinctly, often significantly, more pronounced during river and sea passage than during port stay. In post-hoc analyses, there was a higher level of vibration in the working areas workshop, crew mess room, galley and recreational room during the river passage (for example in the Kiel Canal) compared to sea passages. In the cabin, the maximum vibration was 61 mm/ s^2^.

**Table 3:** Vibration on board depending on the different voyage periods.

Physical influences	Location	*General*	Voyage period		
			*Port stay*	*River passage*	*Sea passage*
**Vibration**^a^, mm/s^2^ median (min-max)	Bridge	31 (4–117)^&^	14 (4–59)	46 (9–117)*	43 (23–65)*
	Office	30 (2–243)^&^	4 (2–10)	32 (6–78)*	58 (7–243)*^#^
	Deck	18 (2–57)^&^	5 (2–10)	19 (6–33)*	30 (5–57)*^#^
	Engine room	62 (3–159)^&^	15 (3–30)	87 (52–159)*	90 (123–148)*
	Engine control room	34 (3–210)^&^	7 (3–16)	35 (9–94)*	54 (9–210)*^#^
	Workshop	37 (1–173)^&^	14 (1–50)	66 (6–173)*	58 (22–88)*^#^
	Crew mess room	26 (3–125)	8 (3–20)	39 (6–125)*	34 (8–85)
	Galley	23 (2–84)^&^	5 (2–8)	37 (10–84)*	30 (6–62)*^#^
	Cabin	22 (4–61)^&^	7 (4–27)	21 (9–32)*	33 (13–61)*
	Recreational room	16 (3–30)	10 (3–30)	20 (16–25)*	17 (12–11)

^&^significant Kruskal-Wallis test in relation to port stay,

*different from “port stay”,

^#^different from “river passage”

^a^presented is the largest vibration of the three axes (most often the z axis)

In order to show the range of physical impacts that the seafarers are normally exposed to during their working routine, the median values (including min to max) of all measured locations were presented. The average air temperature was 24.6 °C (range 12.8–34.9°C), air velocity was 0.28 m/ s (range 0.04–0.78 m/s) and humidity was 26.1% (range 9.8–39.4%) (Table 4). The mean PMV and PPD values were 1.1 (range -0.7–2.7) and 37.0% (range 6.6–94.8%) respectively, with large differences between the vessels. None of the measured climatic parameters or the assessment of thermal comfort differed significantly between the voyage episodes; the PMV or PPD value was slightly lower during the river passage. In post-hoc analyses the air velocity and the humidity were significantly higher in this voyage period compared to port stay.

**Table 4:** Climatic values on board depending on the different voyage periods.

			Voyage period		
Physical influences	Location	*General*	*Port stay*	*River passage*	*Sea passage*
**Climatic parameters**, *median (min-max)*	Air temperature, *°C*	24.6 (12.8–34.9)	24.6 (16.2–33.3)	22.4 (12.8–27.9)	25.1 (15.4–34.9)
	Radiation temperature, *°C*	24.2 (12.9–34.3)	24.5 (17.1–34.3)	22.7 (12.9–28.7)	24.4 (15.5–33.2)
	Wet temperature, *°C*	15.2 (12.9–17.0)	14.4 (12.9–15.9)		15.9 (14.9–17.0)
	Air velocity, *m/s*	0.28 (0.04–0.78)	0.22 (0.05–0.52)	0.42 (0.13–0.67)*	0.29 (0.04–0.78)
	Relative humidity, *%*	26.1 (9.8–39.4)	24.7 (9.8–37.7)	27.6 (20.1–39.4)*	27.1 (15.9–36.5)
	PMV	1.1 (-0.7–2.7)	1.2 (-0.3–2.4)	0.8 (-0.7–1.6)	1.2 (-0.35–2.7)
	PPD, *%*	37.0 (6.6–94.8)	37.3 (6.6–87.3)	31.6 (12.1–55.7)	38.2 (7.4–94.8)
	Engine room				
	PMV	1.2 (-0.7–2.7)	1.4 (-0.3–2.4)	0.7 (-0.7–1.6)	1.2 (-0.4–2.7)
	PPD, *%*	42.4 (6.6–94.8)	45.8 (6.6–87.3)	33.4 (12.1–55.7)	44.9 (7.6–94.8)
	Engine control room				
	PMV	0.8 (0.7–0.8)	0.7		0.8
	PPD, *%*	17.1 (15.6–18.6)	15.6		18.6
	Workshop				
	PMV	0.9 (0.3–2.3)	0.9 (0.4–2.2)	0.9	1.0 (0.3–2.3)
	PPD, *%*	28.6 (7.4–89.1)	27.8 (8.3–84.5)	24.2	30.0 (7.4–89.1)
	Galley				
	PMV	1.3 (0.7–2.1)	1.3 (1.0–1.8)		1.4 (0.7–2.1)
	PPD, *%*	43.4 (15.0–68.9)	43.0 (26.2–68.9)		43.9 (15.0–38.2)

PMV = predicted mean vote; PPD = predicted percentage of dissatisfied

The first 7 mentioned measurements are the average values taken from the above mentioned different areas on board.

*different from “port stay”

The highest temperatures were measured in the engine room (including the separator washing area) and in the galley (during meal preparation). Accordingly, the highest PMV and PPD values were found in these work areas (Table 4). As the fans were often switched off in port, slightly higher heat loads were sometimes recorded in the engine room in this voyage episode.

In respect of the age and size of the examined vessels, no significant differences were observed between the groups below and above the median (year of construction younger than 2006 vs. older than 2006 and smaller vessels vs. larger vessels (gross tonnage >70,000)).

### Person-related measurements in different voyage episodes

The personal noise exposure determined over cumulatively more than 340 hours during specific noise-related occupational activities was particularly pronounced among engine room personnel (96 dB (A)). The deck crew, on the other hand, was exposed to an average level of 83 dB (A) at work, with 15% of the measurements above 85 dB (A) as the upper threshold limit. The noise pollution for the nautical officers was particularly recorded during the monitoring of the loading process on deck and averaged 77 dB (A) (Table 5).

**Table 5:** Task-related noise level of the different occupational groups.

Job tasks	*Cumulative duration of measurement*, *h* : *min*	Task-related noise level (LAeq) in dB (A), *median (min-max)*		
		*Nautical officers (19)*	*Deck ratings (51)*	*Engine room personnel (34)*
**General**		77 (56–91)	83 (50–104)	96 (57–114)
Monitoring of loading process and lashing	57 : 35	80 (62–91)	84 (53–102)	
Gangway watch	51 : 01		80 (50–99)	
Inspection of refrigerated containers	31 : 26		83 (69–98)	86 (70–101)
Control of engine	44 : 25			99 (69–114)
Cleaning work in the engine room	24 : 56			100 (69–114)
Cleaning work on deck	18 : 42		86 (59–101)	
Mooring and unmooring	15 : 13		83 (56–97)	
Repair work on the engine	24 : 56			95 (69–114)
Manual work on board (e.g. knocking off rust)	13 : 25		88 (69–104)	
Coating work on deck	45 : 21		79 (58–96)	
Coating work in the engine room	1 : 46			100 (70–112)
Control and repair work on deck	14 : 53		77 (70–85)	89 (69–105)
Vacuum cleaning	9 : 06		82 (57–94)	

Manual work on board was performed equally by both deck ratings and engine room personnel.

For the engine room personnel, the highest noise exposure was during cleaning or coating work in the engine room, followed by control and repair work on the engine (in some cases also during non-operation of the machine). Activities outside the engine room (e.g., inspection of refrigerated containers, repair activities on deck) were also associated with high noise levels for the engine room personnel.

Noise-intensive manual work on board (e.g. knocking off rust) led to a further high level of noise pollution for the deck and engine room personnel. In addition, cleaning work on deck and the handling of heavy linkages to secure the containers during loading operations (so-called lashing) represent a noise exposure in the range of the upper threshold for the deck workers—assuming a corresponding theoretical exposure over eight hours per day (Table 5).

## Discussion

In the present study, relevant stress due to the physical loads on board was detectable. In view of own observations and the current measurements, it is not surprising that more than 70% of the seafarers had experienced stress due to shipboard vibration or noise. As expected, this was particularly true for the engine room personnel who are exposed to especially high physical impacts at their workplace. This occupational group only stated significantly more often stress at work due to noise compared to the other two working groups. This indicates a good adaptation of the engine room personnel to their job-related impacts.

For the assessment of the personal aural noise exposure during the working shifts, the daily noise exposure level (in relation to 8 h) was determined by dosimetry over several hours and compared with the corresponding limit values. The ancillary local measurements (e.g. on the bridge, in the cabins) usually only required averaging in the minute range because of the stationary nature of the noise sources (e.g., cabin fans, background noise from marine propulsion).

Relevant noise levels were found in the engine room and in the engine control room during port stay. Contrary to the initial assumption of a reduced noise impact on the engine room personnel during port stay, a continuously high noise load was measured without significant noise breaks at the workplace during this voyage period.

The relatively low noise level in the cabins during port stay was surprising. Many seafarers complained in the questionnaires’ free text about significant sleep problems in port as a result of the often noisy container handling, combined with clearly noticeable ship movements. Therefore, this not continuous, but rather impulsive noise impact during container handling seems to be subjectively particularly stressful.

The personal measurements, assuming corresponding effects over eight-hour exposure per day, indicate a noise pollution in the range of the upper threshold values. When staying in a noise area, adequate hearing protection must always be worn to counteract the development of noise deafness. On all ships, appropriate hearing protection was available and was also widely used according to observations by the on-board inspectors. When evaluating the aural effects of the shipboard noise, it should be highlighted that there is a difference between the noise measured at the workplace and the real noise perceived by the seafarers when wearing hearing protection. The seafarers examined used the hearing protection at their workplace during the performance of their routine work so that only minor aural effects are likely. It cannot be excluded, however, that the existing level of noise in the range of the threshold values (over 24 hours per day) can cause extra-aural symptoms (e.g. sleep disturbances or arterial hypertension).

The driving conditions defined in DIN ISO 2923 were not complied with in this study as the focus of the present survey was to measure the seafarers’ stress related to the voyage episode. This study shows that vibration was distinctly more pronounced during river and sea passage than during port stay as expected due to the higher engine power during the both first mentioned voyage episodes. A vibration level above the standard values of DIN ISO 6954 was not detectable within this survey; nevertheless, more than 80% of the seafarers felt stressed by vibration. This is probably due to the fact that seafarers had not experienced sufficient resting and compensating phases in everyday life on board due to the continuous impact of vibration on board for 24 hours a day—combined with a permanent noise load. As a limitation, it should be considered that the performed measurements for vibration do not record ship movement that can also affect the seafarers’ well-being on board. A further general limitation of the present study is the multiple testing problem that may lead to an overestimation of the differences between the voyage episodes.

Concerning climatic influences, an own study including 134 male seafarers revealed that heat in workplaces was regarded 24 times as the most important stressor aboard [1]. In total, there is only little knowledge about levels of exposure to potential health risks in seafarers due to the often temporary and insecure working situation on vessels and the lack of valid long-term information of seafarers’ health impairments that can be related to ship-related physical influences [29].

In the present study, none of the measured climatic parameters or the assessment of thermal comfort differed significantly between the voyage episodes. All climatic measurements on board took place in the North/Baltic Sea area, so that only workplace-related climatic effects were recorded. The impact of high outside temperatures on crews (for example during the ship’s stay in the tropics) was not assessable in this study.

**In summary**, ship crews are exposed to the physical stressors on board for 24 hours a day and 7 days a week. With the exception of the noise in the engine room and partly in the workshop, no extraordinarily high levels of physical impacts were detectable. It should be noted, however, that occupational medical limits are usually based on an eight-hour working day, but seafarers are also exposed to the noise pollution on board during their free and sleeping time. There are no threshold values so far for such load impacts.

In the recreational areas, in particular in the cabin, however, noise exposures above the threshold limits according to MSC 91 were measured on individual ships. This can also reduce the recreational value during the free time on board and lead to fatigue, which has repeatedly been described as a major health problem among seafarers [30–32].
